# Relationship between trajectories of dietary iron intake and risk of type 2 diabetes mellitus: evidence from a prospective cohort study

**DOI:** 10.1186/s12937-024-00925-5

**Published:** 2024-02-02

**Authors:** Ruoting Wang, Yingxin Liu, Lehana Thabane, Ivan Olier, Likang Li, Sandra Ortega-Martorell, Gregory Y.H. Lip, Guowei Li

**Affiliations:** 1 Center for Clinical Epidemiology and Methodology (CCEM), Guangdong Second Provincial General Hospital, Guangzhou, 510317 China; 2 Department of Health Research Methods, Evidence, and Impact, McMaster University, 1280 Main St West, Hamilton, ON L8S 4L8 Canada; 3 Liverpool Centre for Cardiovascular Science, University of Liverpool, Liverpool John Moores University and Liverpool Heart & Chest Hospital, Liverpool, UK; 4 School of Computer Science and Mathematics, Liverpool John Moores University, Liverpool, UK; 5 Danish Center for Health Services Research, Aalborg University, Aalborg, Denmark; 6 Father Sean O’Sullivan Research Centre, St Joseph’s Healthcare Hamilton, 50 Charlton Ave E, Hamilton, ON L8N 4A6 Canada

**Keywords:** Dietary iron intake, Trajectory, Type 2 diabetes mellitus

## Abstract

**Background:**

The association between dietary iron intake and the risk of type 2 diabetes mellitus (T2DM) remains inconsistent. In this study, we aimed to investigate the relationship between trajectories of dietary iron intake and risk of T2DM.

**Methods:**

This study comprised a total of 61,115 participants without a prior T2DM from the UK Biobank database. We used the group-based trajectory model (GBTM) to identify different dietary iron intake trajectories. Cox proportional hazards models were used to evaluate the relationship between trajectories of dietary iron intake and risk of T2DM.

**Results:**

During a mean follow-up of 4.8 years, a total of 677 T2DM events were observed. Four trajectory groups of dietary iron intake were characterized by the GBTM: trajectory group 1 (with a mean dietary iron intake of 10.9 mg/day), 2 (12.3 mg/day), 3 (14.1 mg/day) and 4 (17.6 mg/day). Trajectory group 3 was significantly associated with a 38% decreased risk of T2DM when compared with trajectory group 1 (hazard ratio [HR] = 0.62, 95% confidence interval [CI]: 0.49–0.79), while group 4 was significantly related with a 30% risk reduction (HR = 0.70, 95% CI: 0.54–0.91). Significant effect modifications by obesity (*p* = 0.04) and history of cardiovascular disease (*p* < 0.01) were found to the relationship between trajectories of dietary iron intake and the risk of T2DM.

**Conclusions:**

We found that trajectories of dietary iron intake were significantly associated with the risk of T2DM, where the lowest T2DM risk was observed in trajectory group 3 with a mean iron intake of 14.1 mg/day. These findings may highlight the importance of adequate dietary iron intake to the T2DM prevention from a public health perspective. Further studies to assess the relationship between dietary iron intake and risk of T2DM are needed, as well as intervention studies to mitigate the risks of T2DM associated with dietary iron changes.

**Supplementary Information:**

The online version contains supplementary material available at 10.1186/s12937-024-00925-5.

## Introduction

Type 2 diabetes mellitus (T2DM), accounting for 90–95% cases of diabetes mellitus (DM), is a leading cause of morbidity and mortality [1, 2]. As the population ages, DM is increasingly prevalent worldwide, with an estimated amount of approximately 629 million people living with DM globally by 2040 [3]. Therefore, identifying effective strategies for T2DM prevention is significantly important and remains remarkably challenging.

Previous studies have suggested that dietary factors intricately contribute to the onset of T2DM [4, 5]. Dietary iron is involved in many vital cellular functions including antioxidant defense system function, β-cell metabolism, and insulin secretion [6, 7]. However, excessive body iron stores can accelerate oxidative stress and damage pancreatic islet cells [8]. Several studies evaluating the relationship between dietary iron intake and T2DM risk reported inconsistent conclusions, requiring more evidence for further clarification [9, 10]. A prospective study conducted in China suggested no significant relationship between dietary iron intake and T2DM risk [10]. Nevertheless, another large study indicated that dietary iron intake was nonlinearly associated with DM risk, with an L-shaped relationship among women and a reverse J-shaped relationship among men observed [9]. Moreover, all the previous studies only used the baseline information on dietary iron intake and ignored the dynamic trends, which may also explain the sensitive findings regarding the association between dietary iron and risk of T2DM in the general population. In contrast, the group-based trajectory model (GBTM) that can integrate exposure data collected during a long-term period has been used as an attractive alternative for association studies. By incorporating the exposure information on dynamic changes and potential cumulative effects, the GBTM has been reported to outperform the general practice of using a static baseline measurement [11, 12].

In this study, we aimed to investigate the relationship between dynamic trajectories of dietary iron intake and risk of T2DM based on the data from the nationwide prospective United Kingdom (UK) Biobank study. We hypothesized that the baseline trajectory groups based on dietary iron intake were significantly related to incident risk of T2DM in the general population. We also explored the potential effect modifications to the relationship between trajectories of dietary iron intake and the risk of T2DM.

## Methods

### Study participants

Between 2006 and 2010, UK Biobank enrolled more than 500,000 middle-aged and older participants (39 to 74 years old) with 54% females from the general population. The information was collected through self-completed touch-screen questionnaires (including questions on socio-demographic, lifestyle and health-related factors), physical measurements (including blood pressure, heart rate, and grip strength, to mention a few) and computer-assisted interviews conducted by trained nurses (including questions on medications and operations). All participants provided written informed consent. The study design and data collection details have been reported elsewhere [13].

Dietary information was repeatedly collected for a total of 5 times through a 24-hour dietary assessment questionnaire. To construct the trajectory model, participants with more than 2 missing values across the 5 dietary assessments or without the last dietary assessment were excluded (*n* = 439,039). Participants were also excluded if they had a history of T2DM before all assessments (*n* = 2,339). A history of T2DM was identified by using the information from self-reported illness, medication use, and disease diagnosis codes linkage of the international classification of diseases 9th (ICD-9) and 10th (ICD-10) revisions (STable 1). Subsequently, a total of 61,115 participants were included for analyses in this study (SFigure 1). All participants were followed up from the last dietary assessment until a T2DM diagnosis, death, or the censoring date (March 2017 for England and October 2016 for Scotland), whichever came first.

### Outcome

Our study outcome was time to the incident T2DM event during follow-up, where the incident T2DM was identified by using the information from the combination of ICD-9/10 code, and death registry records (**STable 1**).

### Exposure

Unfortunately, data on the supplemental iron intake could not be available in this study. We therefore focused on examining the relationship between dietary iron intake based on food source alone (food and beverage consumption, excluding any supplements) and risk of T2DM. Detailed information on consumption of foods and drinks in the past 24 h was collected by using the Oxford WebQ that was a validated 24-h dietary recall tool [14], in which the description and accuracy of the dietary assessment at baseline have been reported elsewhere [15, 16]. The first dietary assessment was conducted between April 2009 and September 2010, after which each of the four repeated assessments was collected at a 3–4 month interval [17]. The last dietary assessment at baseline was performed between April 2012 and June 2012. Therefore, the participants had a maximum of five dietary assessments, in which the estimates of dietary iron intake were based on their responses to the dietary questionnaires.

### Other independent variables

Covariates of consideration included age (in years), sex (males and females), ethnicity (white or others), body mass index (BMI; in kg/m^2^), residence area (urban or rural), physical activity (none: 0 MET-mins per week for MVPA [moderate-to-vigorous physical activity]; low: < 600 MET-mins per week; medium: 600–1200 MET-mins per week; and high: ≥ 1200 MET-mins per week), smoking status (current, previous or never), alcohol drinking status (current, previous or never), income (< £ 18,000, £ 18,000 - £ 30,999, £ 31,000 - £ 51,999, £ 52,000 - £ 100,000, or > £ 100,000), socioeconomic status (TDI: Townsend deprivation index), glycated haemoglobin, history of hypertension (yes or no), hypercholesterolemia (yes or no), and cardiovascular disease (CVD) (yes or no). We also collected data on some other dietary variables including dietary intake of total energy, carbohydrates, protein, magnesium, fiber, and saturated fat from the last dietary assessment [9].

### Statistical analyses

The GBTM was used to determine groups with similar dietary iron intake trajectories through the *Proc Traj* command in SAS [18]. We fitted the longitudinal dietary iron intake data in a censored normal model after taking the effects of outliers into account, which was appropriate for continuous data. We tested models with varied number of trajectory groups (from 1 to 5) and different functional forms of cubic, quadratic, and linear terms. The optimal number of trajectories was evaluated by the following composite criteria: (i) the Bayesian information criterion (BIC); (ii) > 5% participants in any single trajectory group; and (iii) confirming visually distinct trajectories [19]. Cubic trajectory models with four dietary iron intake trajectories showed the best fit for the data in our study (STable 2). Each participant was subsequently assigned to the corresponding trajectory group according to the maximum likelihood estimation [20].

Descriptive analysis was performed for continuous variables with mean and standard deviation (SD) and categorical variables with counts and percentages. Chi-square test and one-way ANOVA were conducted for the baseline characteristics by trajectories of dietary iron intake.

We used multiple imputation techniques for the missing data of covariates. Subsequently, Cox proportional hazards models were used to investigate the relationship between four trajectories of dietary iron intake and the risk of T2DM. The findings were reported as hazard ratios (HRs) and 95% confidence intervals (CIs). Two models were conducted, with one adjusted for age, sex, BMI, and dietary intake of total energy (parsimonious model), and the other further adjusted for ethnicity, residence area, smoking status, alcohol drinking status, income, Townsend deprivation index, physical activity, history of hypertension, hypercholesterolemia, CVD, glycated haemoglobin, and dietary intake of carbohydrates, protein, magnesium, fiber, and saturated fat (fully-adjusted model).

We performed several subgroup analyses to explore the potential effect modifications to the relationship between trajectories of dietary iron intake and risk of T2DM, including sex (males vs. females), age (< 65 vs. ≥ 65 years), obesity (yes: BMI ≥ 30 kg/m^2^ vs. no: BMI < 30), history of hypertension (yes vs. no), hypercholesterolemia (yes vs. no), and CVD (yes vs. no). We included the interaction term between the stratifying covariates and trajectories of dietary iron intake in the model to test the potential effect modifications in the fully-adjusted model.

As an exploratory analysis, we conducted the comparisons between the trajectory model (using longitudinal data) and quartile model (using single-point data) based on the Akaike information criterion (AIC) and Harrell’s C-statistic. The quartile model evaluated the association between dietary iron intake as single-point data (the last dietary assessment at baseline) and risk of T2DM, in which we used the lowest quartile with the mean dietary iron intake of 8.1 mg/day as the reference group [9, 21]. We performed three models for the comparisons: Model 1 adjusted for age, sex, BMI, and dietary intake of total energy; Model 2 further adjusted for ethnicity, residence area, smoking status, alcohol drinking status, income, Townsend deprivation index, physical activity, hypertension, hypercholesterolemia, CVD, glycated haemoglobin, and dietary intake of carbohydrates, protein, magnesium, fiber, and saturated fat; and Model 3 with no covariates adjusted for. Moreover, we calculated the mean dietary iron intake for each individual from all the dietary assessments, to conduct another exploratory analysis with the mean grouping model. The mean grouping model evaluated the association between mean dietary iron intake for each individual and risk of T2DM, in which we used the lowest quartile with the mean dietary iron intake of 9.7 mg/day as the reference group. We compared model performances between the trajectory model and mean grouping model based on net reclassification improvement (NRI), integrated discrimination improvement (IDI), AIC and Harrell’s C-statistic, where the NRI and IDI were indicators to assess improvement in risk prediction of the mean grouping model [22, 23].

We further performed a sensitivity analysis with the use of a competing risk model by taking all-cause deaths as the competing events of T2DM. Given the unavailability of specific food sources used to estimate the dietary iron intake, we also conducted four *post hoc* sensitivity analyses to assess robustness of our main findings by further adjusting for (1) red meat (the sum of the servings of beef, lamb and pork; treated as continuous variable); (2) vitamin C and calcium (both treated as continuous variables); (3) use of iron supplement (yes or no); (4) red meat, vitamin C, calcium, and iron supplement.

All tests were two-sided with a significance level of 0.05. We conducted all statistical analyses in SAS software version 9.4 (SAS Institute, Inc., Cary, NC).

## Results

**Fig. 1:**
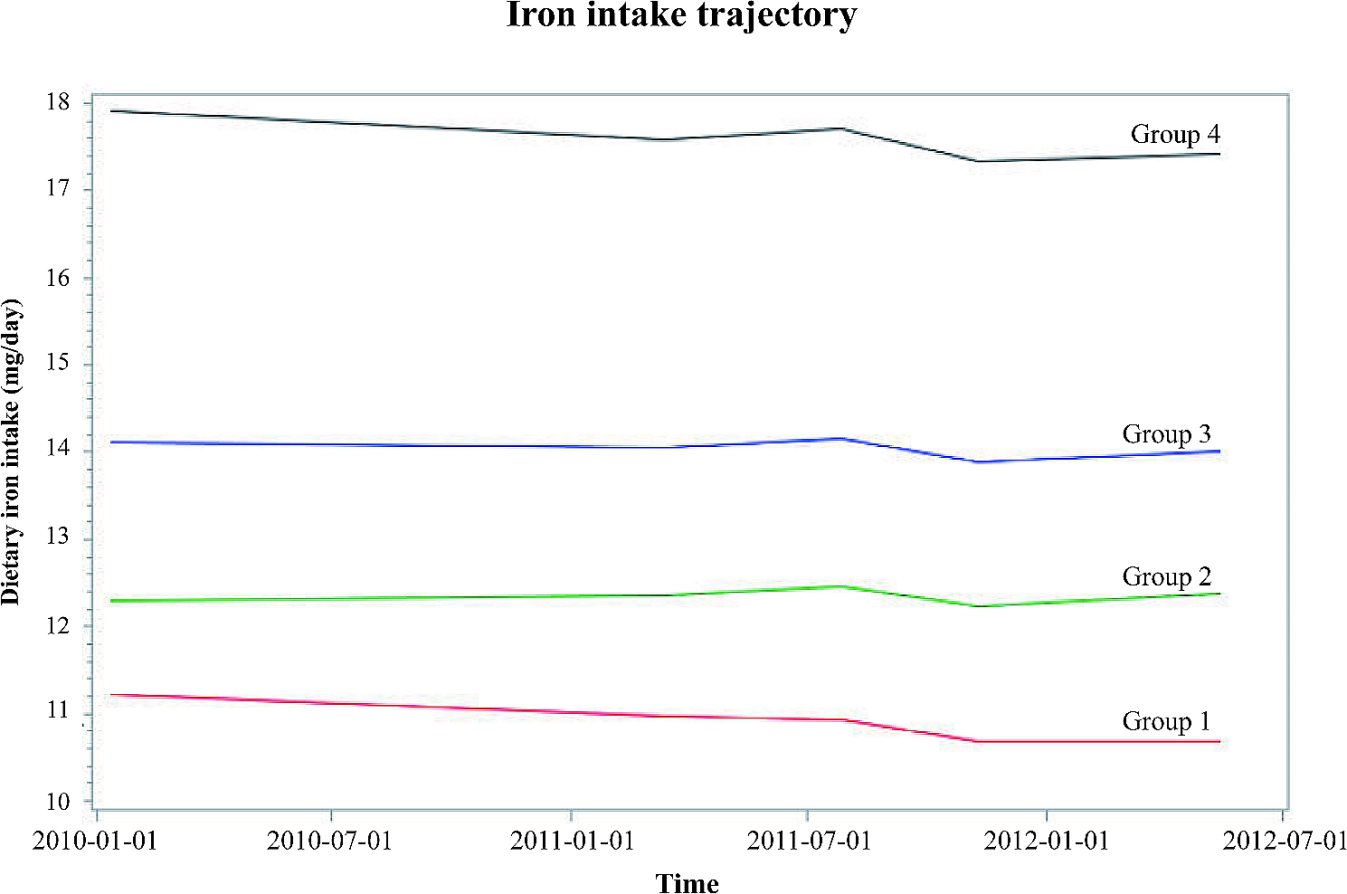
Trajectories of dietary iron intake by follow-up years

The participants (*n* = 61,115) were divided into four dietary iron intake trajectory groups: trajectory group 1 (with a mean dietary iron intake of 10.9 mg/day), 2 (12.3 mg/day), 3 (14.1 mg/day) and 4 (17.6 mg/day) (Fig. 1). The descriptions of baseline characteristics by trajectories were shown in Table 1. Participants in trajectory group 1 were more likely to be females, young, poor, and less likely to have hypercholesterolemia and hypertension compared with other trajectory groups. A significantly higher BMI was also found in trajectory group 1.

**Table 1:** Descriptions of baseline characteristics for the overall participants and by dietary iron intake trajectory groups

Characteristics	Total (*n* = 61,115)	Iron intake trajectory group			
		1 (*n* = 12,976)	2 (*n* = 13,062)	3 (*n* = 19,814)	4 (*n* = 15,263)
Age, mean (SD), y	56.3 (7.7)	55.5 (7.7)	56.1 (7.7)	56.7 (7.6)	56.8 (7.7)
Male, n (%)	26,251 (43.0)	4,066 (31.3)	4,721 (36.1)	8,678 (43.8)	8,786 (57.6)
White ethnicity, n (%)	59,375 (97.2)	12,390 (95.5)	12,673 (97.0)	19,378 (97.8)	14,934 (97.8)
Urban residence area, n (%)	51,348 (84.0)	11,020 (84.9)	10,969 (84.0)	16,604 (83.8)	12,755 (83.6)
Body mass index, mean (SD), kg/m^2^	26.3 (4.4)	26.6 (4.7)	26.5 (4.5)	26.2 (4.3)	26.1 (4.2)
Physical activity, n (%)					
No MVPA	6,281 (10.3)	1,759 (13.6)	1,332 (10.2)	1,886 (9.5)	1,304 (8.5)
Low PA	14,518 (23.8)	3,172 (24.4)	3,183 (24.4)	4,774 (24.1)	3,389 (22.2)
Medium PA	9,623 (15.7)	1,887 (14.5)	2,009 (15.4)	3,258 (16.4)	2,469 (16.2)
High PA	22,313 (36.5)	4,117 (31.7)	4,577 (35.0)	7,273 (36.7)	6,346 (41.6)
Smoking status, n (%)					
Current smoker	3,827 (6.3)	1,080 (8.3)	847 (6.5)	1,052 (5.3)	848 (5.6)
Previous smoker	21,490 (35.2)	4,239 (32.7)	4,537 (34.7)	7,047 (35.6)	5,667 (37.1)
Never	35,690 (58.4)	7,632 (58.8)	7,651 (58.6)	11,681 (59.0)	8,726 (57.2)
Alcohol drinking status, n (%)					
Current drinker	57,606 (94.3)	11,926 (91.9)	12,305 (94.2)	18,788 (94.8)	14,587 (95.6)
Previous drinker	1,688 (2.8)	509 (3.9)	352 (2.7)	503 (2.5)	324 (2.1)
Never	1,789 (2.9)	534 (4.1)	397 (3.0)	510 (2.6)	348 (2.3)
Income, n (%)					
< £ 18,000	7,570 (12.4)	1,771 (13.6)	1,648 (12.6)	2,328 (11.7)	1,823 (11.9)
£ 18,000 - £ 30,999	13,314 (21.8)	2,813 (21.7)	2,864 (21.9)	4,294 (21.7)	3,343 (21.9)
£ 31,000 - £ 51,999	16,105 (26.4)	3,398 (26.2)	3,440 (26.3)	5,234 (26.4)	4,033 (26.4)
£ 52,000 - £ 100,000	14,198 (23.2)	2,810 (21.7)	2,954 (22.6)	4,729 (23.9)	3,705 (24.3)
> £ 100,000	4,506 (7.4)	858 (6.6)	913 (7.0)	1,508 (7.6)	1,227 (8.0)
TDI, mean (SD)	-1.7 (2.8)	-1.5 (2.9)	-1.7 (2.8)	-1.8 (2.8)	-1.7 (2.8)
Use of iron supplements, n (%)	2,087 (3.4)	503 (3.9)	424 (3.3)	632 (3.2)	528 (3.5)
Saturated fat intake, mean (SD), g/day	30.4 (14.9)	25.2 (11.9)	28.8 (26.8)	31.2 (14.7)	35.2 (16.9)
Carbohydrate intake, mean (SD), g/day	253.7 (95.5)	198.0 (71.0)	236.2 (77.8)	261.7 (87.6)	305.6 (106.9)
Protein intake, mean (SD), g/day	82.1 (27.8)	66.6 (22.6)	77.6 (23.7)	84.2 (25.7)	96.3 (30.0)
Magnesium intake, mean (SD), mg/day	350.3 (114.1)	262.3 (75.1)	322.7 (82.8)	361.3 (96.2)	434.6 (122.4)
Fiber intake, mean (SD), g/day	16.2 (6.8)	11.7 (4.8)	14.8 (5.3)	16.8 (6.0)	20.7 (7.3)
Total energy, mean (SD), KJ/day	8854.4 (2843.0)	6945.9 (2047.2)	8268.6 (2253.2)	9121.0 (2545.5)	10632.1 (3066.3)
Glycated haemoglobin, mean (SD), %	5.3 (2.5)	5.3 (2.5)	5.3 (2.5)	5.3 (2.6)	5.3 (2.5)
Cardiovascular disease, n (%)	6,374 (10.4)	1,314 (10.1)	1,301 (10.0)	2,061 (10.4)	1,698 (11.1)
Hypercholesterolemia, n (%)	8,263 (13.5)	1,666 (12.8)	1,774 (13.6)	2,671 (13.5)	2,152 (14.1)
Hypertension, n (%)	32,385 (53.0)	6,664 (51.4%)	6,857 (52.5)	10,627 (53.6)	8,237 (54.0)

SD, standard deviation; MVPA, moderate-to-vigorous physical activity; PA, physical activity; TDI, Townsend deprivation index

During a mean follow-up period of 4.8 years, a total of 677 incident T2DM events were observed: 178 (1.4%) in trajectory group 1, 147 (1.1%) in group 2, 183 (0.9%) in group 3, and 169 (1.1%) in group 4, respectively. Table 2 demonstrates the associations between the four trajectories of dietary iron intake and the risk of T2DM. When compared with group 1, trajectory group 3 was significantly associated with a 38% decreased risk of T2DM from the fully-adjusted model (HR = 0.62, 95% CI: 0.49–0.79), while group 4 was significantly related with a 30% risk reduction (HR = 0.70, 95% CI: 0.54–0.91). This indicated a non-linear relationship between dietary iron intake and T2DM risk, and the lowest T2DM risk was found in trajectory group 3.

**Table 2:** Relationship between dietary iron intake trajectory groups and risk of type 2 diabetes mellitus in parsimonious and fully-adjusted models

Iron intake trajectory group	Mean iron intake, (mg/day)	No. of cases/No. of total participants	Parsimonious model		Fully-adjusted model	
			Hazard ratio (95% CI)	*P*-value	Hazard ratio (95% CI)	*P*-value
**1**	10.9	178/12,976	Ref	-	Ref	-
**2**	12.3	147/13,062	0.79 (0.63, 0.99)	0.04	0.81 (0.65, 1.02)	0.07
**3**	14.1	183/19,814	0.64 (0.52, 0.80)	< 0.01	0.62 (0.49, 0.79)	< 0.01
**4**	17.6	169/15,263	0.70 (0.55, 0.89)	< 0.01	0.70 (0.54, 0.91)	< 0.01

Parsimonious model: adjusted for age, sex, BMI, and total energy

Fully-adjusted model: further adjusted for ethnicity, residence area, smoking status, alcohol drinking status, income, Townsend deprivation index, physical activity, hypertension, hypercholesterolemia, history of cardiovascular disease, glycated haemoglobin, and dietary intake of carbohydrates, protein, magnesium, fiber, and saturated fat

Table 3 presents the subgroup findings for the association between trajectories of dietary iron intake and risk of T2DM. Significant effect modifications by obesity (*p* = 0.04) and history of CVD (*p* < 0.01) were found to the relationship between trajectories and T2DM risk. A lower HR was observed in participants with obesity than those without especially when comparing group 2 with group 1 (with obesity: HR = 0.62, 95% CI: 0.45–0.84; without obesity: HR = 1.09, 95% CI: 0.77–1.52). For the participants without CVD, group 3 had lowest HR compared with group 1 (HR = 0.62, 95% CI: 0.47–0.81), while the lowest HR was found in trajectory group 4 among participants with CVD (HR = 0.41, 95% CI: 0.23–0.73).

**Table 3:** Subgroup analyses for the relationship between dietary iron intake trajectory groups and risk of type 2 diabetes mellitus from fully-adjusted models

Subgroups		No. of cases/No. of total participants	Mean iron intake, (mg/day)	Hazard ratio (95% CI)	P-for interaction
**By sex**					0.53
Females	1	98/8,910	10.8	Ref	
	2	76/8,341	12.3	0.87 (0.64, 1.20)	
	3	64/11,136	14.0	0.53 (0.37, 0.75)	
	4	49/6,477	17.4	0.81 (0.53, 1.25)	
Males	1	80/4,066	10.9	Ref	
	2	71/4,721	12.3	0.77 (0.56, 1.08)	
	3	119/8,678	14.2	0.71 (0.52, 0.98)	
	4	120/8,786	17.8	0.69 (0.49, 0.98)	
**By age**					0.17
< 65	1	137/11,316	10.9	Ref	
	2	118/11,157	12.3	0.88 (0.68, 1.14)	
	3	128/16,593	14.1	0.60 (0.46, 0.78)	
	4	123/12,678	17.7	0.69 (0.51, 0.94)	
≥ 65	1	41/1,660	10.9	Ref	
	2	29/1,905	12.3	0.51 (0.30, 0.85)	
	3	55/3,221	14.0	0.82 (0.52, 1.27)	
	4	46/2,585	17.3	0.86 (0.51, 1.45)	
**By obesity**					0.04
With obesity	1	111/2,551	10.8	Ref	
	2	69/2,381	12.3	0.62 (0.45, 0.84)	
	3	98/3,241	14.1	0.59 (0.44, 0.80)	
	4	83/2,337	17.5	0.59 (0.42, 0.85)	
Without obesity	1	67/10,424	10.9	Ref	
	2	78/10,681	12.3	1.09 (0.77, 1.52)	
	3	85/16,573	14.1	0.67 (0.47, 0.96)	
	4	86/12,926	17.6	0.84 (0.56, 1.25)	
**By CVD**					< 0.01
With CVD	1	46/1,314	10.8	Ref	
	2	45/1,301	12.3	0.93 (0.60, 1.46)	
	3	48/2,061	14.1	0.69 (0.43, 1.10)	
	4	27/1,698	17.7	0.41 (0.23, 0.73)	
Without CVD	1	132/11,662	10.9	Ref	
	2	102/11,761	12.3	0.75 (0.57, 0.97)	
	3	135/17,753	14.0	0.62 (0.47, 0.81)	
	4	142/13,565	17.6	0.80 (0.59, 1.08)	
**By hypercholesterolemia**					0.08
With hyper-cholesterolemia	1	60/1,666	10.9	Ref	
	2	62/1,774	12.3	0.90 (0.62, 1.31)	
	3	69/2,671	14.2	0.78 (0.53, 1.15)	
	4	58/2,152	17.7	0.62 (0.39, 0.97)	
Without hyper-cholesterolemia	1	118/11,310	10.9	Ref	
	2	85/11,288	12.3	0.70 (0.52, 0.93)	
	3	114/17,143	14.1	0.56 (0.42, 0.75)	
	4	111/13,111	17.6	0.70 (0.50, 0.97)	
**By hypertension**					0.16
With hypertension	1	145/6,664	10.8	Ref	
	2	117/6,857	12.4	0.78 (0.61, 1.00)	
	3	151/10,627	14.0	0.63 (0.49, 0.82)	
	4	139/8,237	17.5	0.72 (0.53, 0.96)	
Without hypertension	1	33/6,312	11.0	Ref	
	2	30/6,205	12.2	0.87 (0.52, 1.47)	
	3	32/9,187	14.1	0.53 (0.31, 0.91)	
	4	30/7,026	17.7	0.56 (0.30, 1.06)	

CI, confidence interval; CVD, cardiovascular disease

In the exploratory analyses, with using dietary iron intake from the last assessment at baseline, the parsimonious model showed that quartile 3 dietary iron intake was significantly associated with a decreased T2DM risk when compared with quartile 1 (HR = 0.77, 95% CI: 0.65–0.92), while no significant risk reduction was observed in the fully-adjusted model (Table 4). STable 3 shows the results of comparison between the trajectory model and quartile model. Similar results were observed for Harrell’s C-statistic. A smaller AIC was consistently found for the trajectory model, indicating that the trajectory analysis may show a better fit and outperform the general practice with single-point data. By using participants’ mean dietary iron intake for grouping, the mean grouping model showed that the quartile 3 group (with a mean dietary iron intake of 14.7 mg/day) had the lowest T2DM risk when compared with the quartile 1 group (HR = 0.64, 95% CI: 0.50–0.80; STable 4). STable 5 shows the results of comparison between the trajectory model and mean grouping model. Similar results were observed for Harrell’s C-statistic and AIC, while both NRI and IDI showed negative values, indicating that trajectory model was more precise to predict and quantify the risk of T2DM when compared with the mean grouping model. Several sensitivity analyses yielded similar results to our main findings, as shown in STables 6, 7, 8, 9 and 10.

**Table 4:** Relationship between dietary iron intake from the last assessment at baseline and risk of type 2 diabetes mellitus from the quartile model*

Iron intake from the last assessment at baseline	Mean iron intake, mg/day	Parsimonious model		Fully-adjusted model	
		Hazard ratio (95% CI)	P-value	Hazard ratio (95% CI)	P-value
Quartile 1	8.1	Ref	-	Ref	-
Quartile 2	11.8	0.87 (0.74, 1.03)	0.11	0.96 (0.81, 1.13)	0.61
Quartile 3	14.8	0.77 (0.65, 0.92)	< 0.01	0.83 (0.68, 1.00)	0.06
Quartile 4	20.3	0.86 (0.70, 1.04)	0.12	0.97 (0.77, 1.22)	0.79

* Quartile model evaluated the association between dietary iron intake as single-point data (the last dietary assessment at baseline) and risk of type 2 diabetes mellitus, in which we used the lowest quartile with the mean dietary iron intake of 8.1 mg/day as the reference group

Parsimonious model: adjusted for age, sex, BMI, and total energy

Fully-adjusted model: further adjusted for ethnicity, residence area, smoking status, alcohol drinking status, income, Townsend deprivation index, physical activity, history of hypertension, hypercholesterolemia, cardiovascular disease, glycated haemoglobin, and dietary intake of carbohydrates, protein, magnesium, fiber, and saturated fat

## Discussion

In this study, we identified four trajectories of dietary iron intake, of which trajectory group 3 (with a mean iron intake of 14.1 mg/day) was found to have the lowest T2DM risk with a significant 38% reduction when compared with group 1. Obesity and history of CVD may significantly modify the relationship between trajectories of dietary iron intake and risk of T2DM.

Iron is a critical essential trace element in the diet to form the metal nucleus of many cellular enzymes and play a vital role in diverse metabolic responsibilities including antioxidant defense system function, β-cell metabolism, and insulin secretion [24, 25]. Furthermore, there was evidence suggesting that iron deficiency impaired glucose homeostasis and negatively affected glycemic control [26]. Therefore, sufficient dietary iron intake is required to maintain normal glucose metabolism, which might underlie the significant T2DM risk reduction for trajectory group 3 of dietary iron intake compared with group 1 in this study. Besides, higher body iron stores might destroy cellular macromolecules by catalyzing the formation of free radicals and subsequent the production of reactive oxygen species [27]. Pancreatic beta cells are particularly vulnerable to oxidative stress due to their weak anti-oxidative defense mechanisms, which therefore disturbs insulin secretion and exacerbates insulin resistance [8, 28]. These might explain the smaller T2DM risk reduction in group 4 than in group 3.

In this study, we focused on the longitudinal dietary iron intake with multiple measurements rather than static single-point data or the traditional mean grouping model. The single assessment obtained through the 24-h dietary recall questionnaires may incur recall bias and random error, which would bias the results of association to an unknown extent [29]. In contrast, the trajectory modeling based on the repeated measurements could reliably and accurately reflect the dynamic trend of dietary iron intake, which might provide robust information and have the potential to reduce bias and error when compared to a single-point assessment [30, 31]. Indeed, the trajectory model was a recommended strategy to enhance the statistical phenomenon of ‘Regression to the mean’ that could make natural variation in repeated data get close to real change [30]. Moreover, previous studies had reported that exposure trajectory could serve as a better indicator in assessing the associations than a single measurement by summarizing each group’s trajectories over time in an understandable graphical manner [32–34], and by attempting to reveal a true relationship that may be masked by using single-point data [35]. In terms of the mean grouping model, if participants reported an extremely high (or low) iron intake at one of the dietary assessments, the overall mean iron intake would become unduly high (or low) due to the effect of this potential outlier. Subsequently, the overall mean iron intake would yield inappropriate grouping, compromising the validity of study findings. By contrast, the GBTM was based on the whole trajectory over time by fitting the longitudinal data in a censored normal model after controlling the effects of outliers [18]. With the use of the GBTM to identify four clusters of individuals with similar trajectories, our results showed that the trajectory model consistently outperformed the quartile model and mean grouping model (**STables 3 and 5**), which was in line with previous studies [11, 32, 33]. Thus, understanding the differences in dietary iron intake trajectories between individuals over time may help with accurate assessment of their relationship with T2DM risk, especially given that the trajectory analysis could incorporate the individuals’ temporal changes and potential cumulative effects of dietary iron intake.

Through subgroup analysis, we observed obesity and history of CVD were statistically significant effect modifications to the relationship between trajectories of dietary iron intake and risk of T2DM. The higher T2DM risk reduction found in obese participants might be due to the elevated whole body iron stores compared with the non-obese [36, 37]. The lowest T2DM risk was found in trajectory group 3 among participants without CVD, but in trajectory group 4 among participants with CVD, suggesting a higher dietary iron need for those with CVD. It might be due to iron imbalance in participants with CVD, which can lead to impaired regulation of cardiomyocyte iron metabolism and ferroptosis [38]. Nevertheless, these findings of subgroup analysis required more high-quality evidence to further validate.

One previous study conducted in China suggested that dietary iron intake was nonlinearly associated with DM risk [9], which was in line with our findings of different T2DM risks for the different trajectory groups. However, dietary iron intake with lowest DM risk (25.43 mg/day for males, and 22.05 mg/day for females) was higher in the Chinese study than that in our study (14.1 mg/day). A possible reason might be due to the higher intake for the Chinese study when compared to ours, whereby the lowest dietary iron intake group (17.3 mg/day) in their study had a similar intake to the trajectory group 4 (17.6 mg/day) in our study. The high dietary iron intake for Chinese participants was also observed in another two cross-sectional studies (28.2 ± 12.0 mg/day for men, 23.4 ± 9.5 mg/day for women; 19.6 ± 8.8 mg/day for all participants), indicating the different dietary iron consumptions between the Chinese and UK participants [39, 40]. Besides, another Chinese prospective study suggested no significant relationship between dietary iron intake and T2DM risk [10]. Notably, all these inconsistent study findings may be because all the previous studies focused on dietary iron intake measurement at a single time point [9, 39], without considering the longitudinal course of dietary exposure over time [11].

There was no significant recommendation difference in dietary iron intake from the Food and Nutrition Board between men and women aged over 50 years [41], which was consistent with our result that no significant sex difference was found regarding the dietary iron intake in relation to risk of T2DM. By contrast, the iron intake in trajectory group 3 (14.1 mg/day) with the lowest T2DM risk was higher than recommendation (8 mg/day) [41]. The possible reason might be that the recommended dietary iron intake was established based on the average daily level of intake sufficient to meet the nutrient requirements for most of (97 − 98%) the healthy individuals [42], while in this study we focused on the appropriate intake in relation to reduced risk of T2DM. Therefore, our findings may provide some evidence-based data on dietary iron intake from the perspective of T2DM prevention. Nevertheless, more high-quality evidence is needed to further explore and validate the relationship.

### Strengths and limitations

To our best knowledge, this study is the first attempt to investigate the relationship between trajectories of dietary iron intake and risk of T2DM. The large amount of data with robust analyses supported the validity of our results. While different associations between dietary iron intake and risk of T2DM were consistently reported, findings from this population-based study may help clarify the relationship between dietary iron intake and T2DM risk.

Several limitations need to be noted. Although evidence has suggested that dietary information from touchscreen questionnaires in UK Biobank was reliable, dietary iron intake estimated from self-report may be subject to recall bias and measurement error, thereby weakening the strength of our study findings [15, 43]. Therefore, future studies using objective biomarkers of iron status to further explore and validate the relationship are needed. Moreover, due to the limited data available from the study, nonheme iron intake and heme iron intake could not be distinguished to further explore the association between dietary iron intake and T2DM risk. Although vitamin C and calcium were adjusted for in the sensitivity analysis, no further variables related to iron absorption including fructose, citric acid, phytates, and carbonated beverages could be assessed due to unavailability of these data. We ascertained the incident T2DM according to the ICD codes and death records, thereby potentially underestimating the outcome occurrence in this study. Our results should be interpreted with caution because possible effects of residual and unmeasured confounding could not be completely precluded in an observational study design. Given the low response rate at baseline (5.5%) in the UK Biobank, the generalizability of our findings may be compromised [44].

## Conclusion

We found that trajectories of dietary iron intake were significantly associated with T2DM risk, where the lowest T2DM risk was observed in trajectory group 3 with a mean iron intake of 14.1 mg/day. These findings may highlight the importance of adequate dietary iron intake to the T2DM prevention from a public health perspective. Further studies to assess the relationship between dietary iron intake and risk of T2DM are needed, as well as intervention studies to mitigate the risks of T2DM associated with dietary iron changes.

### Electronic supplementary material

Below is the link to the electronic supplementary material.

Supplementary Material 1

## Data Availability

The data can be available on application to the UK Biobank (www.ukbiobank.ac.uk/).

## Abbreviations

T2DM: Type 2 diabetes mellitus
DM: Diabetes mellitus
GBTM: Group-based trajectory model
BMI: Body mass index
TDI: Townsend deprivation index
MVPA: Moderate-to-vigorous physical activity
CVD: Cardiovascular disease
BIC: Bayesian information criterion
SD: Standard deviation
HR: Hazard ratio
CI: Confidence interval
AIC: Akaike information criterion
NRI: Net reclassification improvement
IDI: Integrated discrimination improvement
