# Supplementary material for: Relationship between trajectories of dietary iron intake and risk of type 2 diabetes mellitus: evidence from a prospective cohort study
Source: Nutr J. 2024 Feb 2;23:15. doi: 10.1186/s12937-024-00925-5 (PMC10835921; doi:10.1186/s12937-024-00925-5)
Supplement: Supplementary file 1 — Supplementary Material 1 [file 12937_2024_925_MOESM1_ESM.pdf]

SFigure 1. Flow diagram showing participant selection for this study

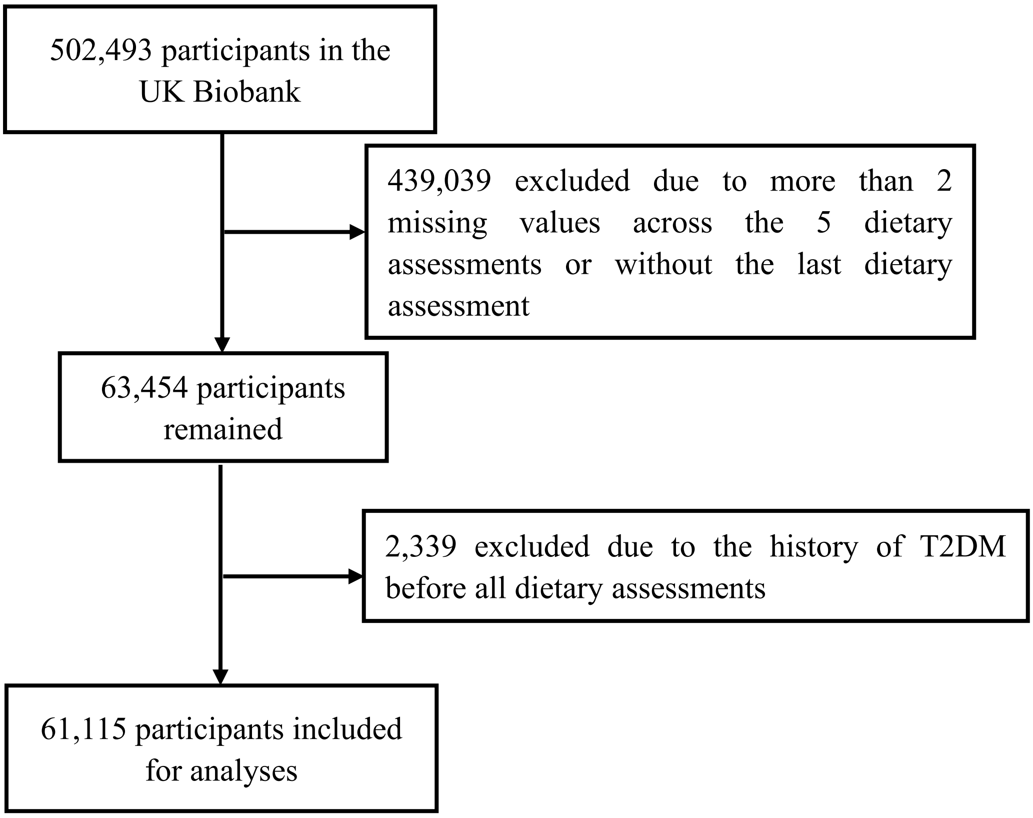

STable 1. Identification of history of and incident type 2 diabetes mellitus in this study

**Data fields**	**Fields' names**	**Data code**
2443[1]	Diabetes diagnosed by physician	-
6153/6177[1]	Use of insulin for diabetes	-
20003[1]	Treatment/medication code	1140883066
20002[1]	Non-cancer illness codes, self-reported	1220, 1222, 1223
41270[2]	Diagnoses - ICD10	E10, E11, E12, E13, E14
41271[2]	Diagnoses – ICD9	250
40001[2]	Underlying (primary) cause of death: ICD10	E10, E11, E12, E13, E14
40002[2]	Contributory (secondary) causes of death: ICD10	E10, E11, E12, E13, E14

ICD = international classification of diseases

[1] for history of T2DM identification only

[2] for both history of and incident T2DM identification

STable 2. Model fit parameter (Bayesian information criterion-BIC) according to number of groups and trajectory shapes

**Number of groups**	**Trajectory shapes***	**BIC (N=61,115)**
2	3 3	-645931.5
3	3 3 3	-643009.8
3	1 3 3	-646688.3
4	3 3 3 3	-642380.7
4	1 3 3 3	-646544.1
4	1 2 3 3	-651734.7
5	3 3 3 3 3	-643546.2

*Trajectory shapes of the best fit model according to a given number of groups; 1 = linear; 2 = quadratic; 3 = cubic.

STable 3. Comparisons of model performances between the trajectory model and quartile model

Model	AIC	C-statistic (95% CI)
**Model 1**		
quartile	25207.18	0.78 (0.77, 0.79)
trajectory	14018.50	0.78 (0.76, 0.79)
**Model 2**		
quartile	16386.70	0.85 (0.83, 0.86)
trajectory	9290.13	0.85 (0.83, 0.86)
**Model 3**		
quartile	26695.56	0.52 (0.51, 0.54)
trajectory	14855.63	0.54 (0.52, 0.56)

Model 1: adjusted for age, sex, BMI, and total energy.

Model 2: further adjusted for ethnicity, residence area, smoking status, alcohol drinking status, income, Townsend deprivation index, physical activity, hypertension, hypercholesterolemia, history of cardiovascular disease, glycated haemoglobin, and dietary intake of carbohydrates, protein, magnesium, fiber, and saturated fat.

Model 3: adjusted for no covariates.

STable 4. Relationship between dietary iron intake (mean dietary iron intake for each individual) and risk of type 2 diabetes mellitus from the mean grouping model*

**Mean dietary iron intake for each individual**	**Mean iron intake, mg/day**	**Hazard ratio (95% CI)**	**P-value**
**Quartile 1**	9.7	Ref	-
**Quartile 2**	12.5	0.73 (0.58, 0.90)	< 0.01
**Quartile 3**	14.7	0.64 (0.50, 0.80)	< 0.01
**Quartile 4**	18.2	0.69 (0.53, 0.89)	< 0.01

*Mean grouping model evaluated the association between mean dietary iron intake for each individual and risk of T2DM, in which we used the lowest quartile with the mean dietary iron intake of 9.7 mg/day as the reference group.

Model was adjusted for age, sex, BMI, total energy, ethnicity, residence area, smoking status, alcohol drinking status, income, Townsend deprivation index, physical activity, hypertension, hypercholesterolemia, history of cardiovascular disease, glycated haemoglobin, and dietary intake of carbohydrates, protein, magnesium, fiber, and saturated fat.

STable 5. Comparisons of model performances between the trajectory model and mean grouping model

Model	Trajectory model	Mean grouping model
**Net reclassification improvement (NRI)**	-0.06<br>(-0.13, 0.02)	
**Integrated discrimination improvement (IDI)**	-0.0003<br>(-0.0007, 0.0001)	
**AIC**	13727.06	13727.47
**C-statistic**	0.8482	0.8468

Both trajectory model and mean grouping model were adjusted for age, sex, BMI, total energy, ethnicity, residence area, smoking status, alcohol drinking status, income, Townsend deprivation index, physical activity, hypertension, hypercholesterolemia, history of cardiovascular disease, glycated haemoglobin, and dietary intake of carbohydrates, protein, magnesium, fiber, and saturated fat.

STable 6. Relationship between dietary iron intake trajectory groups and risk of type 2 diabetes mellitus using competing risk model

Iron intake trajectory group	No. of competing events	Parsimonious model		Fully-adjusted model	
		Hazard ratio (95% CI)	P-value	Hazard ratio (95% CI)	P-value
**1**	225	Ref	-	Ref	-
**2**	230	0.79 (0.63, 0.99)	0.04	0.81 (0.64, 1.01)	0.07
**3**	362	0.64 (0.51, 0.80)	< 0.01	0.62 (0.49, 0.79)	< 0.01
**4**	255	0.70 (0.55, 0.89)	< 0.01	0.70 (0.54, 0.92)	< 0.01

Parsimonious model: adjusted for age, sex, BMI, and total energy.

Fully-adjusted model: further adjusted for ethnicity, residence area, smoking status, alcohol drinking status, income, Townsend deprivation index, physical activity, hypertension, hypercholesterolemia, history of cardiovascular disease, glycated haemoglobin, and dietary intake of carbohydrates, protein, magnesium, fiber, and saturated fat.

STable 7. Relationship between dietary iron intake trajectory groups and risk of type 2 diabetes mellitus by further adjusted for red meat*

**Iron intake trajectory group**	**Hazard ratio (95% CI)**	**P-value**
**1**	Ref	-
**2**	0.81 (0.65, 1.02)	0.07
**3**	0.62 (0.49, 0.79)	< 0.01
**4**	0.70 (0.54, 0.91)	< 0.01

* Model were adjusted for age, sex, BMI, total energy, ethnicity, residence area, smoking status, alcohol drinking status, income, Townsend deprivation index, physical activity, hypertension, hypercholesterolemia, history of cardiovascular disease, glycated haemoglobin, red meat, and dietary intake of carbohydrates, protein, magnesium, fiber, and saturated fat.

STable 8. Relationship between dietary iron intake trajectory groups and risk of type 2 diabetes mellitus by further adjusted for vitamin C and calcium*

Iron intake trajectory group	Hazard ratio (95% CI)	P-value
**1**	Ref	-
**2**	0.81 (0.64, 1.02)	0.07
**3**	0.62 (0.49, 0.79)	< 0.01
**4**	0.70 (0.54, 0.91)	< 0.01

* Model were adjusted for age, sex, BMI, total energy, ethnicity, residence area, smoking status, alcohol drinking status, income, Townsend deprivation index, physical activity, hypertension, hypercholesterolemia, history of cardiovascular disease, glycated haemoglobin, vitamin C, calcium, and dietary intake of carbohydrates, protein, magnesium, fiber, and saturated fat.

STable 9. Relationship between dietary iron intake trajectory groups and risk of type 2 diabetes mellitus by further adjusted for iron supplement*

**Iron intake trajectory group**	**Hazard ratio (95% CI)**	**P-value**
**1**	Ref	-
**2**	0.81 (0.65, 1.02)	0.07
**3**	0.62 (0.49, 0.79)	< 0.01
**4**	0.70 (0.54, 0.91)	< 0.01

* Model were adjusted for age, sex, BMI, total energy, ethnicity, residence area, smoking status, alcohol drinking status, income, Townsend deprivation index, physical activity, hypertension, hypercholesterolemia, history of cardiovascular disease, glycated haemoglobin, iron supplement, and dietary intake of carbohydrates, protein, magnesium, fiber, and saturated fat.

STable 10. Relationship between dietary iron intake trajectory groups and risk of type 2 diabetes mellitus by further adjusted for red meat, vitamin C, calcium, and iron supplement*

Iron intake trajectory group	Hazard ratio (95% CI)	P-value
**1**	Ref	-
**2**	0.81 (0.65, 1.02)	0.07
**3**	0.62 (0.49, 0.79)	< 0.01
**4**	0.70 (0.54, 0.91)	< 0.01

* Model were adjusted for age, sex, BMI, total energy, ethnicity, residence area, smoking status, alcohol drinking status, income, Townsend deprivation index, physical activity, hypertension, hypercholesterolemia, history of cardiovascular disease, glycated haemoglobin, red meat, vitamin C, calcium, iron supplement, and dietary intake of carbohydrates, protein, magnesium, fiber, and saturated fat.
